# Hydrophobic, Mechanical, and Physical Properties of Polyurethane Nanocomposite: Synergistic Impact of Mg(OH)_2_ and SiO_2_

**DOI:** 10.3390/polym15081916

**Published:** 2023-04-17

**Authors:** Zahra Rajabimashhadi, Rahim Naghizadeh, Ashkan Zolriasatein, Sonia Bagheri, Claudio Mele, Carola Esposito Corcione

**Affiliations:** 1 School of Metallurgy and Materials Engineering, Iran University of Science and Technology, Tehran 16846-13114, Iran; 2 Non-Metallic Materials Research Department, Niroo Research Institute, Tehran 1466-5517, Iran; 3 Department of Innovation Engineering, University of Salento, 73100 Lecce, Italy

**Keywords:** polyurethane hybrid composite, magnesium hydroxide, silica, hydrophobicity, hardness, surface roughness

## Abstract

Polyurethane (PU) is one of the most well-known polymer coatings because of its favorable characteristics, which include its low density, nontoxicity, nonflammability, longevity, adhesion, simple manufacture, flexibility, and hardness. However, PU does come with several major drawbacks, among which are poor mechanical properties as well as low thermal and chemical stability, particularly in the high-temperature mode, where becomes gets flammable and loses adhesion ability. The limitations have inspired researchers to develop a PU composite to improve the weaknesses by adding different reinforcements. Magnesium hydroxide, having the ability to be produced with exceptional properties such as flammability, has consistently attracted the interest of researchers. Additionally, silica nanoparticles with high strength and hardness are one of the excellent reinforcements of polymers these days. The hydrophobic, physical, and mechanical properties of pure polyurethane and the composite type (nano, micro, and hybrid) fabricated with the drop casting method were examined in this study. 3-Aminopropyl triethoxysilane was applied as a functionalized agent. To confirm that hydrophilic particles turned into hydrophobic, FTIR analysis was carried out. The impact of size, percentage, and kind of fillers on different properties of PU/Mg(OH)${}_{2}$-SiO${}_{2}$ was then investigated using different analyses including spectroscopy and mechanical and hydrophobicity tests. The resultant observations demonstrated that different surface topographies can be obtained from the presence of particles of different sizes and percentages on the hybrid composite’s surface. Surface roughness allowed for exceptionally high water contact angles, which confirmed the hybrid polymer coatings’ superhydrophobic properties. According to the particle size and content, the distribution of fillers in the matrix also improved the mechanical properties.

## 1. Introduction

Polymer-based coatings are used in a wide range of cases to protect surfaces from corrosive agents like solutions, stress, erosion, wear, and other external variables [1,2]. Pure polymers cannot approach the bulk characteristics of polymer composites reinforced with different types of micro- or nano-fillers [3]. Composites are materials with established borders between fillers that are totally dispersed in a matrix [4]. Depending on the application, the fillers with different sizes, shapes, and properties reinforce the composite qualities [5].

Employing reinforcement fillers, for instance, can enhance the strength of a flexible elastomeric matrix by introducing hard particles and even extra cross-linking sites to the particle–matrix interfaces [6]. Previous research revealed that the polymer’s crosslinking decreased as the interaction between the matrix and the reinforcement became stronger [7]. Raising the filler amount does have two independent and conflicting effects that must be considered: First, there are advantages derived from widening the reinforcement–matrix contact; second, there are disadvantages of lessening the degree of crosslinking in the polymer [8]. A nanocomposite with both traits can be fabricated by adding specific amounts of fillers with properties such as specific morphology, high density, hardness, toughness, and thermal stability to a polymer matrix with poor mechanical and thermal properties [9]. However, the improvement of performance in polymer composites may be greatly influenced by homogenization. It is obvious that when fillers and matrix are inadequately mixed, the particles are not properly dispersed in the polymer. This kind of interaction could cause a reduction in all the properties of a nanocomposite [10].

Polypropylene, epoxy, polyamide, polyvinyl chloride, polystyrene, styrene–butadiene rubber, silicone rubber, and polyurethane (PU) are among those polymers that have been enhanced by inorganic nanoreinforcements [11]. Due to numerous advantages, such as low density, biocompatibility, biostability, nontoxicity, nonflammability, transparency, abrasion resistance, adhesion, antiaging, eas of processing, flexibility, high elongation at break, high impact strength, elasticity, and hardness, polyurethane (PU) is one of the more prominent types of polymer coating [12]. The reaction between the soft component. polyol, and the hard component, isocyanate, results in the two-component PU, which is able to be cured at room temperature [12]. Due to the presence of hard–soft segments in the microstructure, block copolymers found in PU composites have exceptional natural physical characteristics. The soft segment is produced by polyether or polyester polyol, whereas the hard segment is produced by isocyanate and chain extenders [13]. Due to the existence of hard segments, PU exhibits advantageous characteristics such as a high glass transition temperature and a high melting temperature [14]. Nevertheless, PU does have a few significant limitations, particularly in high-temperature working situations, including inadequate tensile strength, poor thermal stability, low thermal and electrical conductivity, and insufficient anticorrosive qualities. In these situations, PU is very flammable and adheres weakly to metal substrates. These limitations have created challenges for PU utilization and have led researchers to make PU composite in order to improve the weaknesses by adding different reinforcements [15]. For example, in the field of environmental issues, the hydrophobicity of coating is extremely substantial. Thus, numerous studies have been conducted aimed at increasing the contact angle of the PU coating [16].

PU nano composites, have considerable benefits above all other typical polymer coatings, such as high adhesion, transparency, and resilience to UV and weathering, and have thus accounted for a significant portion of recent research [17]. Applying inorganic fillers to PU coatings to improve the performance has been the subject of particularly intriguing recent research. To manufacture PU nanocomposite, a variety of nano size reinforcements have been used [18]. The addition of nanofillers to the PU matrix could modify the structure and properties related to the surface qualities, such as resistance to corrosive chemicals, moisture, heat, and hydrophobicity [19]. Compared to typical microparticles, nanoparticles have a larger surface area, greater surface energy, and a tendency to aggregate. Surface modification has therefore been regarded as a substantial solution in the majority of research. Most of the research has concentrated on silane- and fluorine-containing substances. However, using these materials for modification is expensive and harmful to the ecosystem and/or human health [20].

Adding mineral fillers to polymer coatings to improve their performance has long been considered and attractive avenue of research. Most of these studies have been more focused on PU–SO nanocomposites than on PU–MOH nanocomposites and PU–MOH–SO hybrid composites. Nanofillers, for example, ZnO [21,22,23], TiO${}_{2}$ [24,25], and SiO${}_{2}$ [26,27] have been investigated to improve the thermal, electrical, mechanical, surface, and rheological properties of PU resin. The antiaging properties of the PU–ZnO nanocomposite was investigated by Wang. He indicated that samples containing 1%w.t. ZnO could have the best morphological structure after accelerated aging for 500 h [21]. Meanwhile, Saeed focused on a PU-ZnO nanocomposite to investigate antimicrobial properties. During salt spray tests, the growth of bacteria on the sample surface was reduced by the nano filler increment [22]. Moreover, Kumar studied the effect of ZnO addition on the physico-chemical properties of a PU nanocomposite. PU–ZnO illustrated considerable antimicrobial activity compared to pure PU [23]. The PU–TiO${}_{2}$ nanocomposite was studied by Nguyen and his team. Their investigation on mechanical properties demonstrated that the most strength and hardness could be obtained on the optimum content of filler, which was 2%w.t. [24]. Another study was performed on the mechanical properties of PU–TiO${}_{2}$ by Sabzi et al. They examined the effect of filler dispersion and the interaction between matrix and reinforcement. In this regard, amino propyl trimethoxy silane (APTES) was used as a modification agent, and the best tensile strength was obtained at 3%w.t. [25]. The mechanical properties of the PU–SiO${}_{2}$ nanocomposite were also evaluated by Bui. He investigated the effect of filler content on strength, elastic module, and cross-linking via the addition of a 0, 1, 2.5, 5, 7.5, and 10%w.t. reinforcement, and finally, the sample containing 2.5%w.t. was the best one [26]. Song has also investigate nanocomposites, reporting that fine dispersion and interfacial adhesion from 250 to 800 m/µm in 1 and 3%w.t., respectively, increased the wear resistance of PU with the addition of nano-SiO${}_{2}$ [27].

Micronanocomposites, which merge the benefits of micro- and nanoparticles, are a new development in polymer composites. In this regard, Ahmad has proposed a novel method to enhance the stability and reliability of polymers on the premise of a composite structure made of nanoparticles and microparticles. He applied CuO and ZnO fillers, and research results revealed that the merged effects of micro- and nanoparticles could significantly increase electrical resistance and prolong the lifetime of polymer-insulating dielectrics [28]. Zha studied silicone rubber’s thermal conductivity, dielectric capabilities, and mechanical properties by concurrently adding Al${}_{2}$O${}_{3}$ nanoparticles and Si${}_{3}$N${}_{4}$ microparticles. According to the findings of the dielectric breakdown strength test, silicone rubber had a higher breakdown strength if the nanoparticles filled in the spaces left by the microparticles and created conductive channels. The mechanical evaluation of all samples showed that the composite with both micro- and nanoparticles had the greatest tensile strength [29].

Among the studies reviewed, those investigating magnesium hydroxide (MOH) as a fire-resistant, insulating substance and antibacterial agent in nanocomposites were extremely rare. Addition of nanosilica (SO) to PU has appeared as a significant topic in recent studies and could improve a variety of properties of polymers, such as tensile strength, elastic module, strain at break, and hardness. Surprisingly, we could not find any research on PU–MOH nanocomposites, notwithstanding their prominent properties. We thus examined the impact of particle size and MOH concentration on the hydrophobic, physical, and mechanical characteristics of PU nanocomposites due to the paucity of studies on this composite. In addition, we compared this nanocomposite with the PU–MOH–SO hybrid composite to clarify the effect of nano-SO on PU function. According to prior studies, it is challenging to achieve the desirable polymer-reinforcement properties at high filler concentrations, and 5%w.t. has been indicated as the appropriate amount. Therefore, PU–MOH nanocomposites were fabricated via the drop casting method with the addition of the 1, 3, and 5%w.t. reinforcement. Eventually, the effect of the MOH amount on the different properties was investigated using FESEM, AFM, hardness, tensile strength, and contact angle tests. The best results were found in 3%w.t. As a consequence, two hybrid composites were also evaluated with this filler content to study the effect of MOH particle size and the combination with nanosilica: PU–HMOH (containing nano- and micro-MOH powder) and PU-HSO (containing nano-MOH and SO powder).

The remainder of this article is organized as follows: raw materials, sample preparation, and test analysis are all covered in depth in Section 2, Materials and Methods. Section 3 presents the results and discussion based on the characterization and analysis. Finally, conclusion remarks are provided in Section 4.

## 2. Materials and Methods

The exceptional qualities of PU make it ideal for resistant coatings. However, it has poor mechanical and physical characteristics, such as low hydrophobicity, hardness, and compressive strength. In this study, we used the obvious benefits of MOH and SO to address the above-mentioned challenges. This research’s main novelty is the addition of MOH with different particle sizes on nano- and microscales as well as the application of SO to PU coatings. Along with this approach, we evaluated how these fillers affect the hybrid composite in terms of contact angle, surface roughness, and mechanical properties. The materials and methods, including raw materials, the process of producing composites, and test analysis, are covered in detail in the following sections.

### 2.1. PU Composites Fabrication

**Figure 1:**
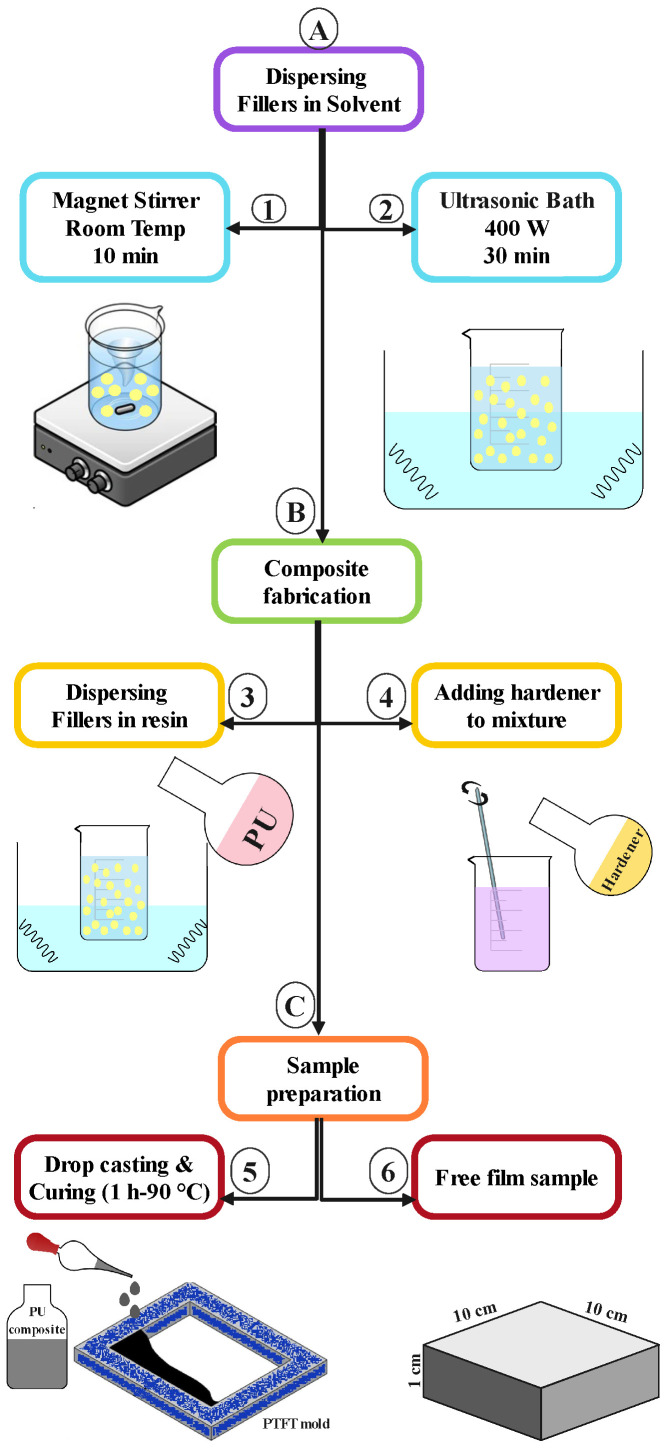
Fabrication of the PU composites via the drop casting method.

The method for fabricating the composite samples is shown in Figure 1. In order to obtain fine particle dispersion in PU matrix, we used functionalized powder. MOH nanopowder (nMOH) was synthesized with the hydrothermal method which we investigated in a previous study with 75 nm plate particles [9]. However, the remainder of the raw materials were purchased from commercial companies whose characteristics are given in Table 1. Composite fabrication was started by filler dispersion in xylene. This solution was mixed completely for 10 min on a magnet stirrer and subjected to ultrasound for 30 min. Then, the filler solution was added to the PU resin and blended. An ultrasonic bath was used again to achieve a favorable homogeneity. After 30 min, the PU hardener was slowly added into the mixture. The final two steps involved mixing by hand and with a glass stirrer to avoid the formation of bubbles in the mixture. Eventually, the final mixture was smoothly poured into a 10 × 10 cm PTFT mold (drop casting method). Composite samples were cured at 90 °C for 1 h.

The chemical information of the samples is listed in Table 2. Density was obtained through the rule of mixtures with specified weight percent and density of components in the final composite (Equation (1)). According to past references, the ratio of NCO:OH derived from the ratio of polyacrylate (resin) to polyisocyanate (hardener) was considered 1:2 to obtain the best qualification of polymer [17]. Then, according to the mold volume, the amount of each component was calculated to fabricate a free film with a 1 cm of thickness (Equations (2) and (3)). In the following equations, D, X, M, and V stand for density, weight percent, molecular weight, and volume, respectively.

(1) $$D_{\mathrm{Composite}\;}={\left(\frac{X_{\mathrm{Filler}\;1}}{D_{\mathrm{Filler}\;1}}+\frac{X_{\mathrm{Filler}\;2}}{D_{\mathrm{Filler}\;2}}+\frac{X_{\mathrm{Resin}\;}}{D_{\mathrm{Resin}\;}}+\frac{X_{\mathrm{Hardener}\;}}{D_{\mathrm{Hardener}\;}}\right)}^{-1}$$

(2) $$M_{\mathrm{Composite}\;}=D_{\mathrm{Composite}\;}\times V_{\mathrm{mold}\;}$$

(3) $$M_{\mathrm{Component}\;}=X_{\mathrm{Component}\;}\times M_{\mathrm{Composite}\;}$$

### 2.2. PU Composite Characterization

For the purpose of filler characterization, field Emission scanning electron microscopy analysis (FESEM) and Fourier-transform infrared spectroscopy (FTIR) were performed. FESEM analysis was completed using a SEM made by MIRA3TESCAN-XMU, Kohoutovice, Czech Republic.

FTIR spectroscopy was used to evaluate the chemical groups of hydrophobic agents on the surfaces of fillers using using the FT/IR-6000 FTIR spectrometer made by FT/IR-6000 FTIR spectrometer, Cremella (LC), Italy. In order to study the PU composite, field emission scanning electron microscopy analysis (FESEM), atomic force microscopy analysis (AFM), and hydrophobicity (static contact angle) and mechanical tests (hardness and tensile strength) were performed. FESEM was used to determine the distribution of fillers in the PU composite microstructure. AFM was carried out to evaluate the surface roughness at the nanometer scale at a 5 × 5 -micron area. The static contact angle was measured to examine the effect of different fillers on the hydrophobicity of the PU composite. The hardness of the PU composites was assessed using a Type A Durometer (Teclock GS-709 N, Japan) in accordance with ISO868. This instrument measures the hardness of a substance, usually a rubber, elastomer, or polymer. Harder materials provide better indentation resistance, as indicated by higher numbers on the scale (Shore A). The interaction between stress and strain is revealed by the material’s stress–strain curve. This was achieved by systematically loading a test zone, measuring the deformation, calculating the stress and strain based on the results, and using dumbbell-shaped samples with a length of 50 mm and a tensile testing equipment (ISO 37 standard, gauge length = 20 mm, strain rate = 10 mm/min).

## 3. Results and Discussions

### 3.1. Filler Characterization

**Figure 2:**
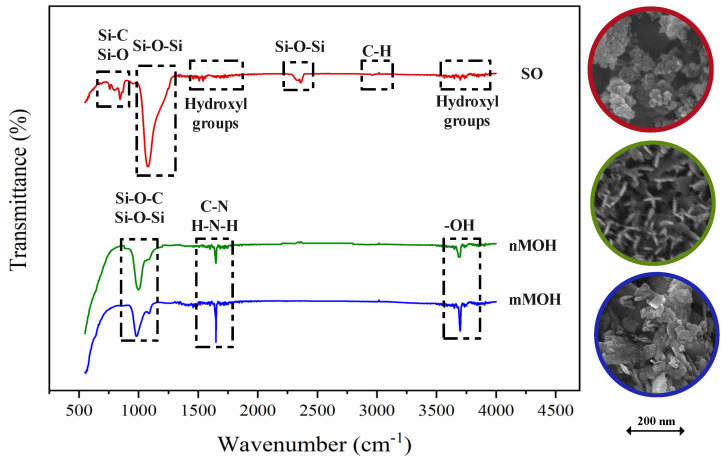
FESEM and FTIR analysis of fillers. While the mMOH powder was had no morphology, nMOH and SO were observed with a nanoplate and nanospherical morphology, respectively.

Figure 2 demonstrates the characterization of the nMOH, mMOH, and SO fillers. According to the FESEM images, nMOH which was studied in another paper [9], has a vertical orientation and plate shape. The mMOH presents agglomerated micron particles, but SO was formed in a spherical shape. FTIR spectroscopy shows the hydrophobic agent on the filler surfaces. For both nano- and micro-MOH, the peak at 3698 cm${}^{-1}$, identified in all samples, refers to the -OH bond in the MOH structure. The peaks that are observed at about 1620 cm${}^{-1}$ in the FTIR spectrum correspond to the stretching mode of C-N and H-N-H as well as the wide peaks around 1050 cm${}^{-1}$, which belong to the stretching vibration of the Si-O-Si or Si-O-C bonds. These two peaks indicate that the long-chain silane groups on the MOH surface come from APTES as a functionalized agent [30,31]. For the SO filler, HDMS was used as a hydrophobic agent. The peak at 2962 cm${}^{-1}$ of the absorbance spectrum is for the stretching vibration of the C–H groups. Therefore, it can be concluded that the –CH${}_{2}$ and –CH${}_{3}$ groups were present on the surface of SO due to surface agents. Many noisy peaks at 3500 to 4000 cm${}^{-1}$ and 1400 to 1500 cm${}^{-1}$ characterize the presence and the bending vibration of the hydroxyl groups. The broad and sharp peaks at 1000 to 1200 cm${}^{-1}$ and 500 cm${}^{-1}$ show the stretching vibration of the Si–O bond [32].

### 3.2. PU Composite

#### 3.2.1. Microstructure Characterization

**Figure 3:**
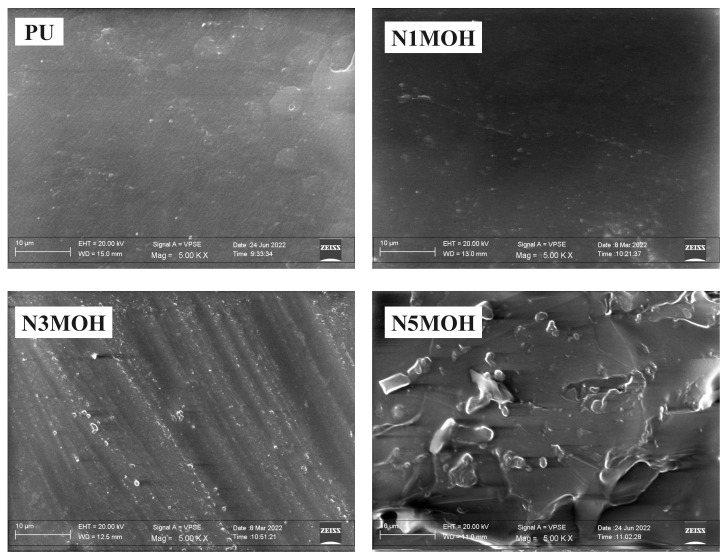
FESEM (5000×) of PU composites with different nMOH contents. While the PU sample demonstrated a smooth surface, the nanocomposites presented more roughness in the microstructure.

**FESEM** Figure 3 compares the microstructure of the PU composite with different amounts of nMOH at 5000× magnification. The surface of the pure sample (PU) is smooth and uniform in texture, whereas the addition of nMOH causes the surface to seem rougher in the nanocomposite samples. This roughness in microstructure is one of the most effective factors in surface properties such as the hydrophobicity [33]. Furthermore, by examining the FESEM images in more depth, it is possible to determine the influence of additional parameters, even as the dimension, morphology, quantity, and dispersion of the nanofillers in the PU matrix [1,2]. The effect of the morphology could be ignored due to the fact that all nanocomposite samples in Figure 3 contain 1, 3, and 5%w.t. of nMOH. By comparing the surface microstructure of nanocomposites with different filler contents, it can be seen that, remarkably, the surface of N1MOH is less rough. This lower degree of roughness could be related to the low amount of nMOH nanofillers in the PU matrix as compared to that of the other samples (1%w.t.). On the other hand, because there is a small amount of nanofiller in the composite, the particles are properly distributed throughout the polymer matrix, and this caused a homogeneous and uniform microstructure without any agglomerated particles [11]. Using the same approach and increasing the amount of nano filler by 3 and 5%w.t. in the PU matrix made the surface of the N3MOH and N5MOH composites rougher. Indeed, the surface roughness continues to excess with increasing filler percentage so that in sample N5MOH, the surface roughness is quite clear. As indicated in all of other relevant research, filler concentration, which passes through the critical level, causes a nonuniform distribution of fillers and changes the surface morphology [14]. By observing the sample surface, we can see that there is no cracking, which indicates the optimal quality of sample fabrication.

**Figure 4:**
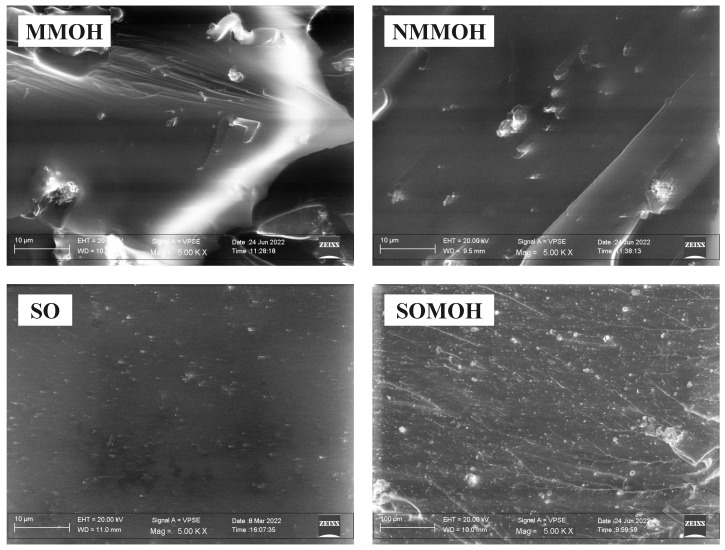
FESEM (5000×) of PU hybrid composites with different fillers including nMOH, mMOH, and SO. While the hybrid and nanocomposite sample (SOMOH) demonstrated a uniform microstructure with homogeneous roughness, the hybrid and microcomposites presented more roughness and heterogeneity in microstructure.

Figure 4 also shows the comparison in the surface microstructure of the PU hybrid composite with two different fillers including mMOH and SO at 5000× magnification. The surface roughness produced in hybrid composites is higher than that in the nanocomposites [34], as it is obvious in the surface microstructure of MMOH, NMMOH, SO, and SOMOH samples. The MMOH sample had a more irregular and heterogeneous microstructure than did all the composites as a result of the addition of the mMOH microfiller to the formulation. Because the abrasive particles did not produce a uniform roughness, we can surmise that the inhomogeneity may cause a considerable reduction in the microcomposite properties. In contrast, the SO was softer compared to all the samples due to the presence of a very fine silica nanoparticles. The simultaneous addition of nMOH and mMOH in the NMMOH hybrid composite created a relatively rough microstructure that was heterogeneous in some parts. However, by applying two nMOH and SO nanoparticles in the SOMOH hybrid nanocomposite, a uniform microstructure with homogeneous roughness was obtained. This theory has been published in a study on hybrid micronanocomposites, where it was shown that by adding more fillers with different size scales, nanoparticles could be inserted into the spaces between the microparticles to act as a bridge-like link [3].

#### 3.2.2. Hydrophobicity Analysis

**Static contact angle**

**Figure 5:**
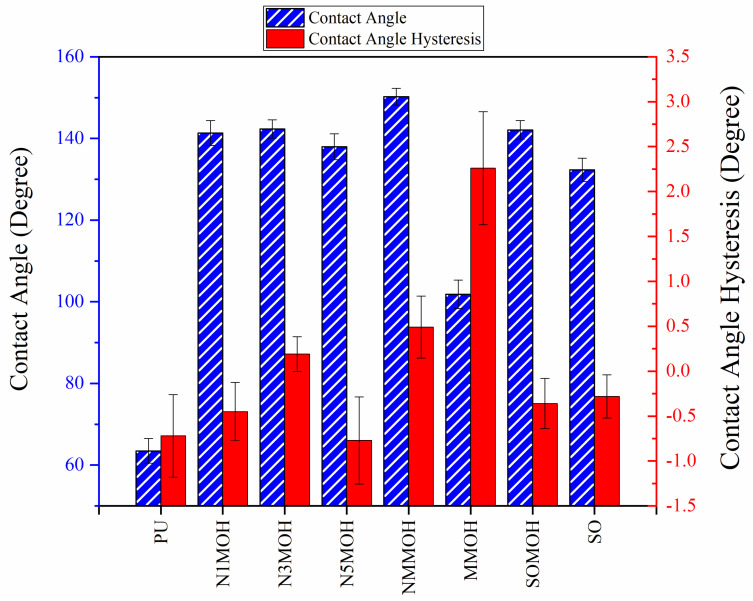
Alterations in the hydrophobicity of the PU composites. The hydrophobicity increased if the filler added to the matrix resulted in a uniform and favorable surface roughness in the composite.

The influence of applying nMOH, mMOH, and SO fillers on the hydrophobicity of PU composite was examined by measuring the contact angles of a water droplet (0.5 cc) with the surface. Table 3 presents the static contact angles of samples, and the comparison of hydrophobicity is available in Figure 5. A broader contact angle was offered by all composite samples—nano, micro, and hybrid—as compared with the PU sample. As anticipated from the FESEM images, the added fillers in the matrix caused surface roughness, and as a consequence, the contact angle improved via the addition of micro- and nanoparticles.

**Figure 6:**
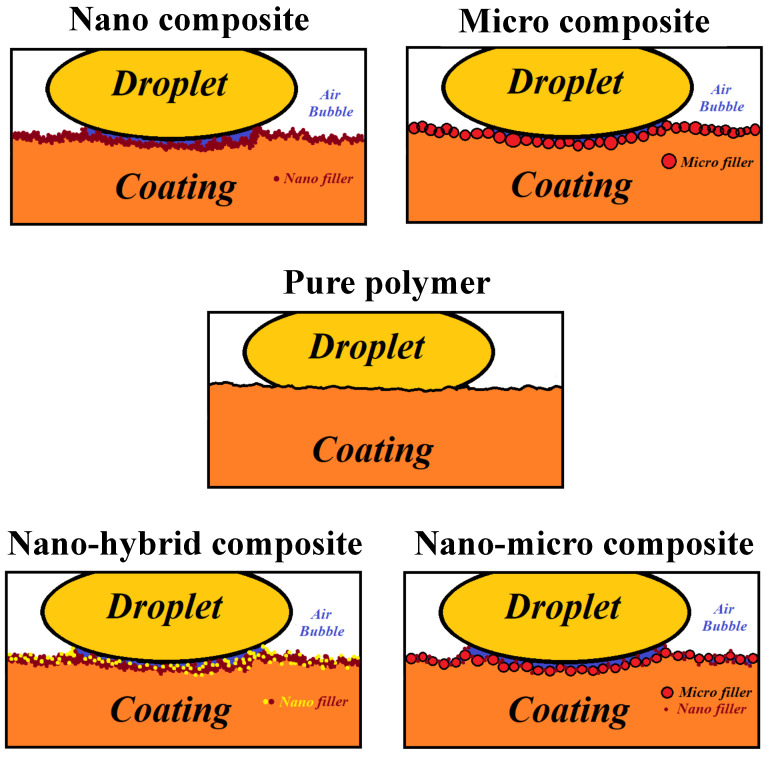
Schematic of the proposed the effect of adding nano- and microfillers on the ups and downs of the surface, increasing surface roughness, water drop shape on the coating (wettability), contact angle, and hydrophobicity.

A surface is considered hydrophobic in general if a drop of water does not tend to stick to the substrate. Here, the contact angle exceeds 90°. On the other hand, a surface will be hydrophilic and have a contact angle below 90° if a drop of water leads to a spread over the substrate [17]. In terms of its wettability, PU is not totally hydrophilic and not totally hydrophobic. The range of its potential applications will increase if it can change from a more hydrophilic to a more hydrophobic characteristic. Hence, making it a superhydrophobic compound is extremely difficult [16,34]. As seen in Figure 6, the average contact angle of 63.5° ± 0.5 in PU increased to 141.4° ± 0.3, 142.3° ± 0.2, and 137.9° ± 0.4 in the presence of different amounts of nMOH in N1MOH, N3MOH, and N5MOH, respectively (1, 3, and 5%w.t.). Several studies have shown that adding nanofillers to PU may increase surface roughness, which in turn may improve the contact angle. Several researchers have shown that the hydrophobicity of a polymer matrix can be improved by the addition of nanoparticles and low surface energy components [29]. According to the literature, applying fillers in the composites with an optimal percentage could enhance the properties, whereas less or more content can result in a nonfavorable impact [33,35]. For this reason, in the N5MOH sample, despite the presence of nanofiller in the matrix, the percentage of particles exceeding the optimal limit caused an unfavorable surface roughness to occur and the contact angle to decrease.

According to Figure 5, the average contact angle for the NMMOH, MMOH, SOMOH, and SO samples were 150.2° ± 0.3, 101.8° ± 1.2, 142.8° ± 0.3, and 132.3° ± 0.2, respectively. Hybrid micronanocomposite samples presented wider contact angles than did nanocomposites. Indeed, the simultaneous addition of the mMOH and nMOH micro- and nanofillers in the NMMOH hybrid composite demonstrated the best hydrophobic properties. In adding micro particles alone, the contact angle was greatly reduced. Numerous researchers have demonstrated that an appropriate polymer matrix’s hydrophobicity can be increased by including nanoparticles and low surface energy elements. Meanwhile, the addition of fillers in different size scales causes smaller particles to be placed in the empty space between larger particles and to create a greater roughness on the nanoscale [21,22]. The presence of SO with and without nMOH inside the PU matrix obtained enhancement in the contact angle similar to that of the N3MOH sample. This means that the small difference in particle size has less effect on the surface roughness. Of course, SOMOH the hybrid composite is more hydrophobic than is SO nanocomposite due the same reason of a contact angle increment occurring in the NMMOH sample.

Researchers have been typically in two independent parameters: the amount and size of nanoparticles on the polymer surface. Meanwhile, the existence of different nano- or microfillers in the same sample can produce a different surface roughness, which will effect the hydrophobicity. The difference in the contact angle of the pure PU sample and the nano-, micro-, and hybrid composite samples could be explained by the hydrophobicity of the lower chemical energy elements resulting in a low gravitational force between the surface and the drop of the water [11]. In order to improve hydrophobicity, the polyurethane’s molecular structure either needs to be modified during synthesis, or hydrophobic additives must be physically added to the PU’s chemical components. Because it is more difficult and expensive, the first method cannot be used to produce superhydrophobic PU surfaces on a large scale [17,18]. Subsequently, by extending the specific surface area of hydrophilic thin film, such as PU, and then modifying it with low-energy components, such as functionalized nanofillers, the contact angle and hydrophobicity could be enhanced [19].

The lotus theory proposes that there are multiple ups and downs on the outer surface of coatings and a certain quantity of air remains between these spaces when a drop of water touches the surface, enhancing the contact angle. In fact, at hydrophobic surfaces, the droplet–coating interface can be replaced with a droplet–air interface and significantly decrease surface tension. These ups and downs could change the shape of the water droplet on the surface. The shape of a droplet/air interface is obtained by the Young equation:

(4) $$\;\gamma_{\mathrm{SG}}-\gamma_{\mathrm{SL}}-\gamma_{\mathrm{LG}}\cos\theta_{C=0}$$

In this equation, $\gamma_{\mathrm{SG}}$, $\gamma_{\mathrm{SL}}$, $\gamma_{\mathrm{LG}}$, and $\theta_{C}$ point to the surface–air interfacial energy, surface–droplet interfacial energy, droplet–air interfacial energy, and equilibrium contact angle, respectively. The theoretical description of the contact is based on the study of the thermodynamic equilibrium between the three phases—liquid (L), solid (S), and gas or vapor (G). According to the mechanism proposed in Figure 6, surface roughness does have a significant effect on surface wettability and contact angle. Whether the droplet fills in surface roughness or leaves air spaces determines the influence of roughness on the hydrophobicity [16,17].

#### 3.2.3. Surface Roughness

**AFM**

**Figure 7:**
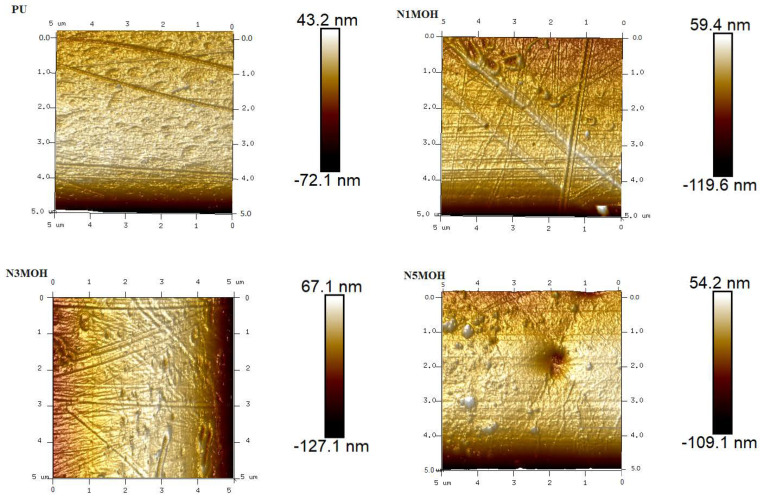

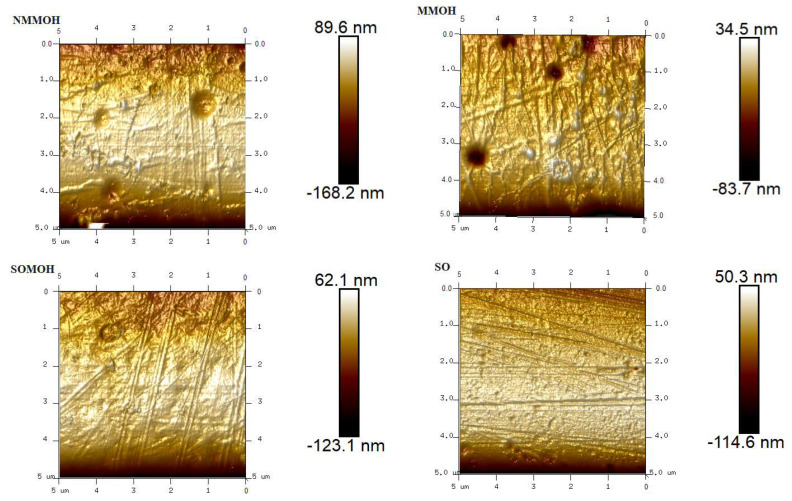
AFM analysis and the surface roughness changes in the PU composite samples produced via the addition of nano- and microfillers.

Figure 7 shows the AFM images of the PU and nano-, micro-, and hybrid composites containing nMOH, mMOH, and SO fillers. Obviously, the results obtained from FESEM images (Figure 3) and contact angle (Figure 5) are established with AFM images. The two-dimensional protrusions on the surface cause the contact angle to increase remarkably. Hydrophobicity could be enhanced due to lingering air bubbles and the lack of the wetting of the surface by the water droplet. The contact angle between air and water droplets on the surface increases when a surface has a roughness in the micronanometer scale. Indeed, the capillary force between the water droplet and the surface swiftly diminishes, and eventually, the water droplet remains a spherical shape on the surface. On a smooth surface without any nano- or microscale roughness, it is almost impossible to obtain a contact angle of more than 120° through simple surface chemical modification. Thus, a key element of hydrophobic coatings is surface roughness. The Wenzel and Cassie–Baxter regimes both explain the contact angle enhancement driven by surface roughness and topography. The Cassie–Baxter regime is more affected by the sticking air bubbles in the ups and downs of the surface, which has a significant impact on the contact angle and may raise hydrophobicity even though the surface roughness increases the contact angle in both regimes [4,5,12].

**Figure 8:**
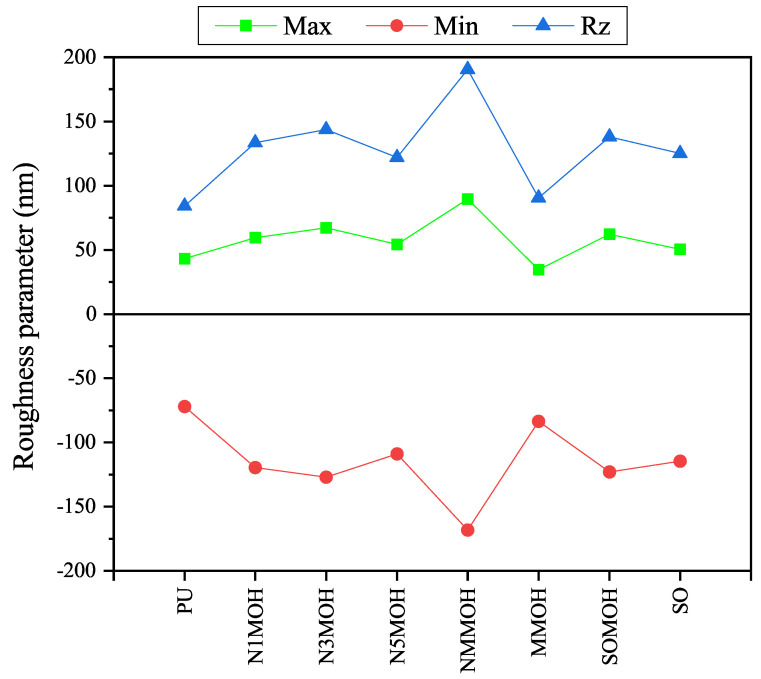
Comparison of surface roughness and Rz parameters for the nano-, micro-, and hybrid composites. This graph confirms the results obtained from the contact angle and hydrophobicity test.

The surface roughness produced by the nano-, micro-, and hybrid compounds is depicted in Figure 8. The parameters Rz (the square root of the sum of the ups and downs) confirm the increment in surface roughness of the PU coating via the addition of the nano- and microfillers. It is demonstrated that the surface roughness of the micronanocomposite NMMOH sample is the highest amount. In contrast, the Rz factor for the microcomposite MMOH sample is almost close to the PU pure sample. This diversity in surface roughness is the reason for the difference in contact angle.

#### 3.2.4. Mechanical Properties

**Hardness**

**Figure 9:**
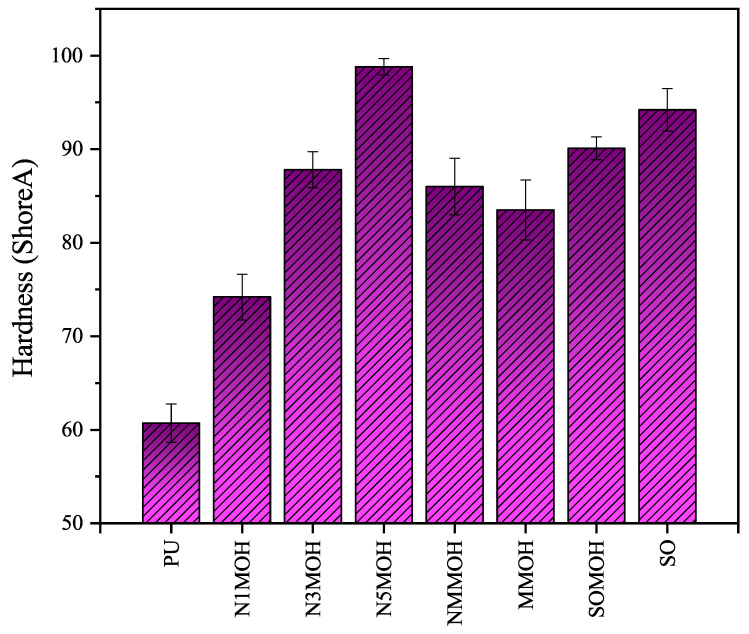
Comparison of hardness values on the Shore A scale of the nano-, micro-, and hybrid PU composite. Hardness was improved due to the presence of the SnMOH, mMOH, and SO hard particles inside the soft PU matrix.

Figure 9 displays the hardness variation of the pure PU sample with the nano-, micro-, and hybrid composites. This test illustrated that hardness was enhanced from an initial value of 60 Shore A for the pure PU sample to a maximum level of 99 Shore A for the nanocomposite N5MOH sample with the most filler content (5%w.t.). This increment refers to the presence of the nMOH rigid fillers inside the flexible PU matrix [13]. Since SO is a much harder nanoparticle than is nMOH, its presence in the SO sample with 3%w.t. caused the hardness almost to be close to that of the N5MOH sample (94 Shore A). Hardness is defined as a low strain modulus and can be improved by gradually adding more filler [15]. There was no significant difference in the hardness of the N3MOH and NMMOH composites (87 and 88 Shore A) in accordance with the constant nanoparticle content in these samples (3%w.t.) and the dependence of hardness on the amount of filler in PU matrix. Although the MMOH sample had the same amount of filler (83 Shore A), it had less hardness, which is related to the agglomeration of microfillers and its heterogeneous microstructure. On the other hand, SOMOH hybrid nanocomposite was harder (90 Shore A) despite having 3%w.t. filler, which is due to the simultaneous addition of SO and nMOH to PU matrix.

**Tensile properties**

**Figure 10:**
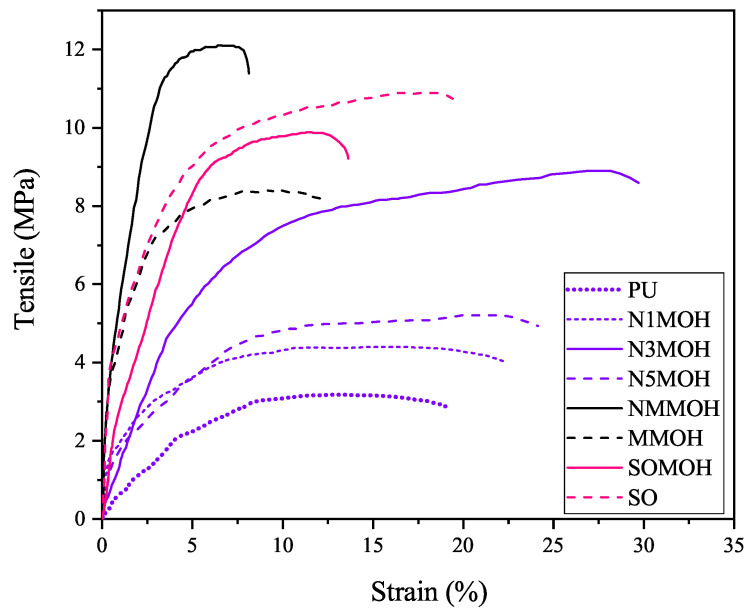
Stress-–strain curves of the nano-, micro-, and hybrid PU composite samples.

**Figure 11:**
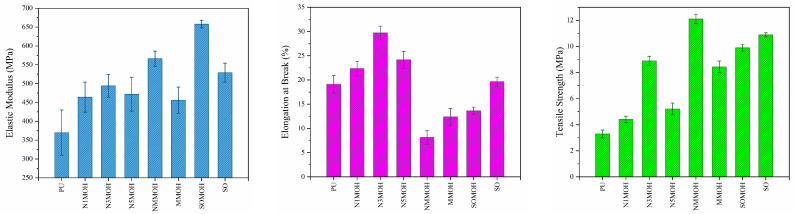
Tensile strength, elongation at break, and elastic modulus variations of the nano-, micro-, and hybrid PU composite samples.

Stress–strain curves of pure PU and the nano-, micro-, and hybrid composites are shown in Figure 10, while the mechanical properties are presented in Figure 11. The best mechanical properties including tensile strength, maximum elongation at break, and elastic modulus were not observed in a given sample. The mechanical strength of MNMOH had the highest amount of the samples due to the better interaction of the nMOH and mMOH fillers with PU chains. On the other hand, the elastic module of the SOMOH nanohybrid composite provided the most value compared the other samples. This could be related to the SO nanofiller with the highest hardness [25]. According to Figure 11, all composites display better mechanical properties than does pure PU. With desirable filler dispersion in the polymer matrix, both the rate of load transmission at the filler–polymer interface and the composite strength could be increased [26]. It can also be seen that small but numerous pinning sites can prevent cracks from growing and expanding. Nanoparticles function as physical cross-linkers that reduce PU chain mobility while enhancing tensile modulus and strength. The tensile modulus and strength will decrease while the strain increases because weak zones and cracks will develop at high nanoparticle concentrations, and the strain stress will be distributed nonuniformly [27]. Elongation at a break is often reduced via the addition of micro- or nanofillers in the polymer matrix. For this reason, in samples containing hard SO nanoparticles (SO and SOMOH) or mMOH microparticles not well dispersed in the matrix (NMMOH and MMOH), a shorter elongation at the breaking point have been reported. The kind, amount, and strength of the link between the filler and the matrix determine the mechanical characteristics of strength and fracture strain [16,34].

## 4. Conclusions

PU is one of the most functional polymer coatings, representing a considerably attractive target for improvement via nanofillers. Very little research has examined MOH as a fire-resistant, insulating material and antibacterial agent in nanocomposites. Despite this, many studies have focused extensively on the addition of SO to PU, which can enhance a variety of polymer properties such as tensile strength, elastic module, strain at break, and hardness, among other. Hence, this study’s main purpose was to investigate the synergistic effect of three different fillers, nMOH, mMOH, and SO, on several properties of PU coatings, including hydrophobicity, surface roughness, and mechanical properties. Consequently, the PU–MOH nanocomposites were fabricated with drop casting method with different nMOH contents, including 1, 3, and 5%w.t. Subsequently, the effect of the nMOH amount on different properties of PU coating was evaluated using FESEM, AFM, hardness, tensile strength, and contact angle tests. The most favorable results were found in the N3MOH nanocomposite. Therefore, two hybrid composites were also studied with this filler content to evaluate the effect of the MOH particle size and the combination with nanosilica: PU-HMOH (containing nano- and micro-MOH powder) and PU-HSO (containing nano-MOH and SO-powder). The presence of nano- and microparticles of MOH simultaneously created a favorable roughness on the surface of the coating and led to a 150% increase in the contact angle of the NMMOH sample compared to PU. Furthermore, this micronanocomposite sample reached the best tensile strength, four times higher than that of the pure sample. The reason for this improvement in mechanical properties is due to the better link formation between fillers and the PU chains in the matrix. Ultimately, this study demonstrated that the synchronic addition of fillers on the nano- and microscale sizes, along with the fabrication of hybrid composites, could improve the hydrophobic and mechanical properties of the coating to a much greater extent than could the nanocomposites.

## Figures and Tables

**Table 1:** Materials used for PU/Mg(OH)${}_{2}$ fabrication.

Material	Chemical Formula	Manufacturer (Cast No.)	Role	Particle Size	Density
nMOH	Mg(OH)${}_{2}$	Synthesized	Nano filler	75 nm	2.34 g cm${}^{-3}$
mMOH	Mg(OH)${}_{2}$	Merck (1309-42-8)	Micro filler	6 µm	2.34 g cm${}^{-3}$
SO	SiO${}_{2}$	Fadak (Hydrophobic grade)	Nano filler	27 nm	2.65 g cm${}^{-3}$
Polyacrylate	Tacryl 765X	Taak Resin Kaveh	Resin	-	1 g cm${}^{-3}$
Polyisocyanate	Desmodur N75	Covestro AG	Hardener	-	1.06 g cm${}^{-3}$
Xylene	C${}_{8}$H${}_{10}$	Dr. Mojallali (1330-02-07)	Solvent	-	-

**Table 2:** The chemical information of the PU composite samples.

Sample Code	nMOH (%w.t.)	mMOH (%w.t.)	SO (%w.t.)	PU resin (%w.t.)	Hardener (%w.t.)	Xylene (%w.t.)
PU	0	0	0	67	33	30 (Extra)
N1MOH	1	0	0	66.5	32.5	30 (Extra)
N3MOH	3	0	0	65	32	30 (Extra)
N5MOH	5	0	0	64	31	30 (Extra)
NMMOH	1.5	1.5	0	65	32	30 (Extra)
MMOH	0	3	0	65	32	30 (Extra)
SOMOH	1.5	0	1.5	65	32	30 (Extra)
SO	0	0	3	65	32	30 (Extra)

**Table 3:** Static contact angle of the PU composites. The presence of different fillers including nMOH, mMOH, and SO had a direct effect on how the drop was placed on the surface.

Sample Code	Static Contact Angle (°)	Water Drop Image	Sample Code	Static Contact Angle (°)	Water Drop Image
PU	63.5 ± 0.5	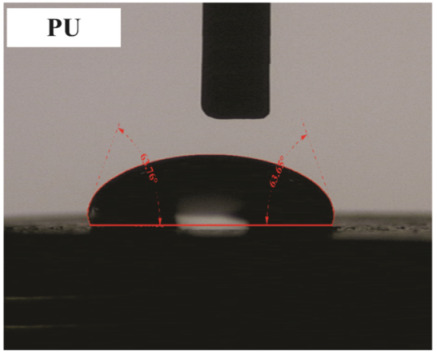	NMMOH	150.2 ± 0.3	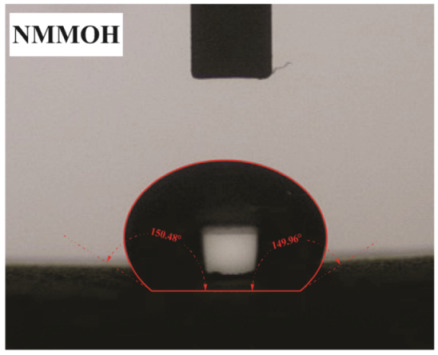
N1MOH	141.4 ± 0.3	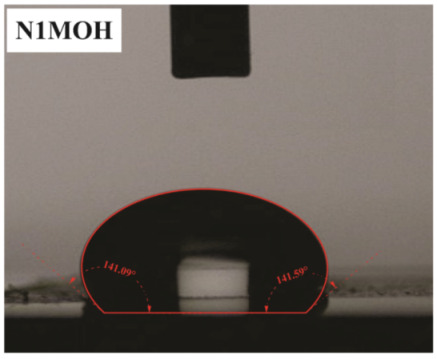	MMMOH	101.8 ± 1.2	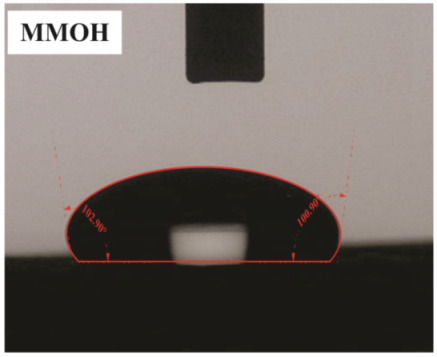
N3MOH	142.3 ± 0.2	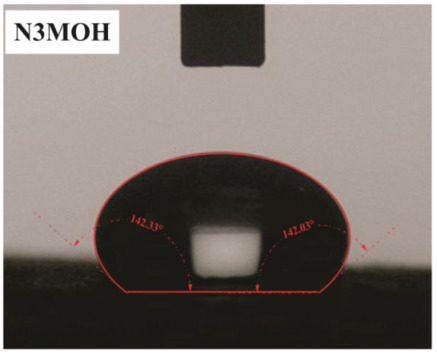	SOMOH	142.8 ± 0.3	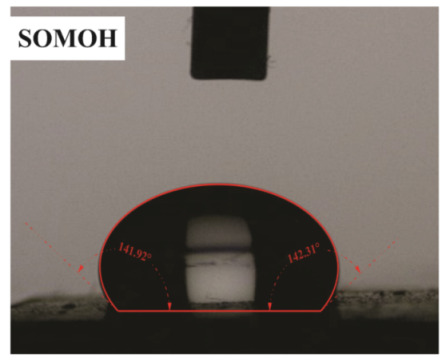
N5MOH	137.9 ± 0.4	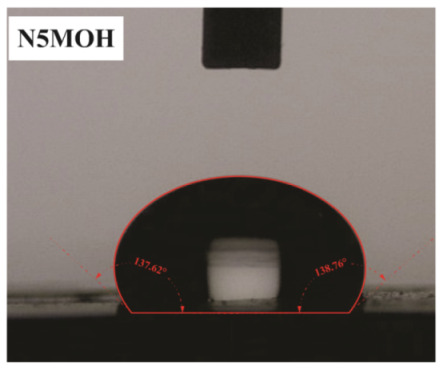	SO	132.3 ± 0.2	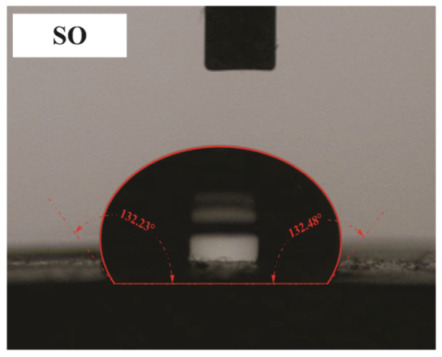

## Data Availability

All data generated or analyzed during this study are included in this published article.
